# Minimizing Adverse Environmental Impact: How Murky the Waters

**DOI:** 10.1100/tsw.2002.186

**Published:** 2002-06-07

**Authors:** Reed W. Super, David K. Gordon

**Affiliations:** Riverkeeper, Inc., 25 Wing & Wing, Garrison, NY 10524, USA

**Keywords:** adverse environmental impact, cooling-water intake structure, entrainment, impingement, power plant, 316(b), aquatic ecology, fisheries, densitydependence, surplus production, compensation theory

## Abstract

The withdrawal of water from the nation’s waterways to cool industrial facilities kills billions of adult, juvenile, and larval fish each year. U.S. Environmental Protection Agency (EPA) promulgation of categorical rules defining the best technology available to minimize adverse environmental impact (AEI) could standardize and improve the control of such mortality. However, in an attempt to avoid compliance costs, industry has seized on the statutory phrase “adverse environmental impact” to propose significant procedural and substantive hurdles and layers of uncertainty in the permitting of cooling-water intakes under the Clean Water Act. These include, among other things, a requirement to prove that a particular facility threatens the sustainability of an aquatic population as a prerequisite to regulation. Such claims have no foundation in science, law, or the English language. Any nontrivial aquatic mortality constitutes AEI, as the EPA and several state and federal regulatory agencies have properly acknowledged. The focus of scientists, lawyers, regulators, permit applicants, and other interested parties should not be on defining AEI, but rather on minimizing AEI, which requires minimization of impingement and entrainment.

### INTRODUCTION

Steam-electric–generating facilities use water for cooling and, in particular, to condense the steam used to drive the turbines. Some power plants withdraw hundreds of millions or billions of gallons of river, lake, or ocean water per day[1]. These plants and all other significant users of cooling water harm and kill large numbers of fish and other aquatic biota[2] through impingement[3] and entrainment[4]. In the early 1970s, a number of well-publicized massive fish kills occurred at U.S. power plants, such as the Brayton Point Power Station in Mt. Hope Bay, Massachusetts, which killed an astonishing 164.5 million menhaden and river herring in just one day, July 2, 1971[5]. In 1972, the U.S. Congress mandated in the Federal Water Pollution Control Act (Clean Water Act or CWA) that cooling-water intake structures (CWISs) use the best technology available (BTA) for minimizing such adverse environmental impact (AEI)[6].

Unlike other sources of degradation to aquatic ecosystems controlled under the 1972 CWA amendments, however, CWISs have uniquely avoided nationally uniform limitations. Instead, regulation of CWISs has long been relegated to *ad hoc* determination by individual permit writers exercising best professional judgment. This lack of categorical standards has resulted in uneven and conflicting regulation as well as enormous, unnecessary aquatic mortality, which runs contrary to the goals of the CWA and the direct mandate of section 316(b). The individualized assessments have typically relied on narrow and inaccurately applied population models and have ignored further impact on ecosystem health.

[1] The nation's largest user of cooling water, the Salem Nuclear Generating Station in New Jersey, withdraws 3.024 billion gallons of water each day from Delaware Bay.

[2] For brevity, this paper uses the word "fish" to denote "fish at all life stages and other aquatic biota," unless the context clearly indicates otherwise.

[3] Impingement is the trapping of adult or larger juvenile fish against an intake's screening devices.

[4] Entrainment is the drawing of small fish, eggs, larvae, and other organisms through a CWIS into the plant's cooling system and heat exchanger.

[5] U.S. Environmental Protection Agency, *Development Document for Best Technology Available for the Location, Design, Construction and Capacity of Cooling Water Intake Structures for Minimizing Adverse Environmental Impact*, 1976 at p. 9, table I-3. EPA reported that the fish were "mangled." *Id.* Mt. Hope Bay forms the northeast arm of the Narragansett Bay estuary.

[6] Clean Water Act section 316(b); 33 U.S.C. § 1326(b), which provides:

> Any standard established pursuant to [Section 301 or Section 306 of the Act] and applicable to a point source must require that the location, design, construction, and capacity of cooling water intake structures reflect the best technology available for minimizing adverse environmental impact.

## DISCUSSION

### Congressional Intent in Enacting Section 316(b) to Minimize AEI

Congress enacted section 316(b) as part of the CWA amendments of 1972 in response to a number of well-profiled fish kills at power plants in the early 1970s. For example, in addition to the Brayton Point incident, the P.H. Robinson plant in Galveston Bay, Texas, impinged more than 7 million fish in 12 months in 1969 and 1970, and the Indian Point No. 1 nuclear facility on New York's Hudson River killed 1.3 million fish over a 10-week period[7]. In the late summer of 1971, more than 2 million dead menhaden clogged the screens at the Millstone plant in Niantic Bay, Connecticut[8]. In fact, during debate over the CWA, Senator Buckley cited with approval two newspaper articles reporting a decision of the Atomic Energy Commission (AEC) to require Consolidated Edison (Con Ed) to install closed cycle cooling at Indian Point[9]. The articles noted that the plants withdrew massive amounts of water from the Hudson River, entraining thousands of organisms per minute, and that the AEC had ordered Con Ed to stop removing such large volumes of water from the River and to install cooling towers in order to abate these massive fish kills[10]. Public concern over these and other incidents prompted Congress to add section 316(b) to the CWA[11].

[7] Clark and Brownell, *Electric Power Plants in the Coastal Zone: Environmental Issues* (1973), p. V-8, table V-B. See also *New York Times Abstracts*, May 24, 1972, p. 94, col. 1 ("alleged 'massive' killing of fish at [Con Ed's] No. 2 nuclear-power plant at Indian Point on the Hudson River"); *New York Times Abstracts*, March 1, 1972, p. 77, col. 3 ("more than 100,000 fish have been killed in last wk [at Indian Point]").

[8] Clark and Brownell, *Electric Power Plants in the Coastal Zone: Environmental Issues* (1973), p. V-8, table V-B. See also *New York Times Abstracts*, August 16, 1972, p. 41, col. 1 ("massive fish kill in Apr at Millstone Point nuclear power complex").

[9] Senate Committee on Public Works, *A Legislative History of the Water Pollution Control Act Amendments of 1972*, 93d Congress, 1st Session, at 196-197, 1973. See also *In the Matter of: Carolina Power & Light Company (Brunswick Steam Electric Plant)*, U.S. Environmental Protection Agency, Decision of the General Counsel, EPA GCO 41 (June 1, 1976) at fn. 10.

[10] *Id.*

[11] See e.g. Yost and Thomas, "Science in the Courtroom", in Barnthouse et al., ed., *Law, Science and the Hudson River Power Plants,* American Fisheries Society Monograph 4, 1988:

> Originally [section 316] only included subsection (a), which provided the utilities with a way to avoid cooling towers if they could convince EPA that they were not needed. That section only applied to the *thermal discharge* from a power plant. Concerned citizens, environmentalists, marine and aquatic biologists, and other scientists alerted Congress to the dangers presented to the aquatic community by the mortality induced by entrainment and impingement. Thus, in a last minute amendment, Congress added subsection (b), which was directed at the *intake* portion of the power plant's generating processes.

*Id.* at 299 (emphasis in original). Judge Yost presided over the Hudson River power plant hearings in the late 1970s.

The structure of the Act indicates how Congress intended the section 316(b) "best technology available" standard for minimizing AEI to be implemented. The Act prohibits all discharges of pollutants to waters of the U.S. except as permitted in a National Pollutant Discharge Elimination System (NPDES) permit[12]. EPA established industry-wide, nationally uniform, technology-based control standards, without regard to site-specific water parameters (such as receiving water quality) to govern the setting of individual NPDES permit limitations[13]. Once established by EPA, these national, technology-based standards must be incorporated into every individual NPDES permit issued nationwide. The goals of technology-based standards are to bring all facilities up to state-of-the-art pollution control as quickly as possible (sometimes referred to as "technology forcing") and to ensure national consistency in NPDES permit limitations[14].

Congress chose the NPDES permitting program as the vehicle for minimizing AEI by making the provisions of § 316(b) applicable to any facility containing a point source[15]. Section 316(b)'s explicit cross-reference to sections 301 and 306 further clarifies that cooling-water intake standards are an integral component of the NPDES technology-based regulations[16]. As a result, EPA must promulgate national technology-based regulations specifying BTA for minimizing AEI, as it does for effluent limitations under sections 301 and 306[17]. This integration, along with the spare and direct "best technology available" mandate, clearly indicates Congressional intent that EPA set nationwide technology-based standards for CWISs in the same fashion as for chemical

[12] 33 U.S.C. § 301(a); see also 33 U.S.C. § 1342 (NPDES program).

[13] See 40 C.F.R Parts 402–699. In waters that violate ambient quality standards, a more restrictive set of limitations may apply. See 33 U.S.C. §§ 1312, 1313, 40 C.F.R. Parts 130–131.

[14] A primary objective of Congress in implementing nationally applicable standards was to avoid the "race to the bottom," which commonly occurred in the absence of uniform national effluent limitations prior to the adoption of the Act, where states would compete to attract and maintain industries by relaxing control requirements. See Hines, "Controlling industrial water pollution: Color the problem green," 1968, *9 B.C. Indus. and Comm. L. Rev.* **553**, p. 573; Grad, *Treatise on Environmental Law*, v.2, § 303[a-1].

[15] 33 U.S.C. § 1326(b).

[16] Section 301 mandates the "best available technology" for existing sources while the section 306 new source performance standard must reflect the "best available demonstrated control technology." 33 U.S.C. §§ 1311(b)(2)(A), 1316(a)(1). Congress' use of substantially similar statutory language in Section 316(b) underscores its intent to incorporate that section's limitations into the categorical standards of sections 301 and 306:

> [T]he regulations issued under § 316(b) are…closely related to the effluent limitations and new source performance standards of §§ 301 and 306… It bears emphasis that § 316(b)…requires § 301 and § 306 standards to deal with cooling water intake structures….[The] regulations [are] issued at least in part under the same statutory sections, some of which limit intake structures, others, effluent discharges.

*Virginia Electric and Power Company v. Costle (VEPCO),* 566 F.2d 446, 450 (4th Cir. 1977); see also *Cronin v. Browner*, 898 F.Supp. 1052, 1059 (S.D.N.Y. 1995).

[17] See Consent Decree, October 10, 1995, *Cronin v. Browner*, No. 93 Civ. 0314 (AGS), as amended March 27, 2000; *VEPCO*, 566 F.2d at 450.

pollutants. Such standards apply to permittees across the board, despite potential claims by regulated parties that their individual discharges — or cooling-water intakes — do not cause substantial ecological impact.

On December 18, 2001, as required by Congress in section 316(b) and the District Court in the *Cronin v. Browner* consent decree, EPA promulgated BTA regulations for CWISs at new facilities. [See 66 Fed. Reg. 65256 (December 18, 2001), hereinafter referred to as "Phase I Rule."]

### PLAIN MEANING OF THE PHRASE "ADVERSE ENVIRONMENTAL IMPACT"

In its plain and ordinary meaning, the phrase "adverse environmental impact" refers to any negative effect on any aspect of the environment, and clearly encompasses nontrivial aquatic mortality. The dictionary definitions of each word in the phrase bear that out. "Adverse" means "unfavorable or antagonistic"[18]. "Environment" means "the air, water, minerals, organisms and all other external factors surrounding and affecting a given organism at any time"[19]. "Impact" means "influence; effect"[20]. Thus, anything that affects the environment in a negative way has an adverse environmental impact.

In a gambit to create an additional technical and procedural hurdle to effective regulation, industry insists that permitting agencies must pointedly define AEI at some threshold level of ecological damage for each individual application[21]. The contention is inconsistent with the structure of the CWA and the statutory language. There is no need or sound reason to precisely define a level of acceptable impact, or ascertain the level of unnecessary killing before destabilization of population, to implement the statutory requirement to "minimize adverse environmental impact"[22]. This unremarkable and (in most cases) relatively simple injunction is directly analogous to the modifier "control" for discharge standards under section 306 of the Act. Both terms define the respective purpose of the technology (in each case required to be the

[18] Random House Webster's College Dictionary, 1999. In the environmental law context, "adverse" is often used as the opposite of "beneficial."

[19] Random House Webster's College Dictionary, 1999.

[20] *Id.*

[21] July 11, 2000, letter from Utility Water Action Group Cooling Systems Committee Chair David Bailey to OMB Office of Information and Regulatory Affairs Deputy Administrator Don Arbuckle, at 2, attached to July 11, 2000 letter from Kristy A.N. Bulleit to EPA Office of Science and Technology Director Geoffrey Grubbs. See also Comments of the Utility Water Action Group on EPA's Proposed Section § 316(b) Rule for New Facilities and ICR No. 1973.01, November 9, 2000 ("UWAG Phase I Comment"), at 53-72.

[22] Industry and other parties have at times framed the debate as whether the CWA addresses "population level" impacts or "individual" fish. See e.g UWAG Phase I Comment at 58-68. The dichotomy is false and unhelpful. Neither the Act nor fisheries advocates maintain a serious interest in the survival of an individual fish, or even more absurdly, an individual larva. *Cf.* UWAG Phase I Comment at 60. This obvious fact does not, however, justify requiring demonstration of measurable population decline or, even worse, "unacceptable risk to the population's ability to sustain itself," *id.* at 65, as a threshold for regulating the cooling-water intake at all. Notably, as a further threshold, UWAG would require a showing that the population decline "is attributable to the operation of the cooling water intake structure." *Id.*

best available): to control pollutant discharges under section 306 and to minimize ecological damage due to cooling-water withdrawals under section 316(b). In other words, they supply the answer to the question, "Best technology for what?", which is basic to the meaning of the respective sections. There is nothing in the statutory language that requires a separate determination of some supposed level of AEI, or a self-defeating assessment of how much unnecessary killing it will take to push a population into long-term decline, before a permitting agency can minimize the damage.

Notably, section 316(b) contains no threshold adjective such as "*significant* adverse environmental impact." That stands in marked contrast to the similar language Congress used 3 years earlier in the National Environmental Policy Act of 1969 (NEPA). NEPA requires preparation of an environmental impact statement (EIS) for every major federal action "significantly affecting the human environment"[23]. Thus, although both statutes address environmental impact, in NEPA the significance of the impact is a threshold requirement for the EIS requirement. Conversely in section 316(b), the threshold requirements are that there must be a CWIS and a point source. Additionally, in its Phase I Rule, EPA adopted a 2 million gal/day *de minimis* withdrawal threshold, below which the national new facility regulation would not apply, to avoid burden to "smaller operations that may face issues of economic affordability and are therefore more appropriately addressed on a case-by-case basis using [best professional judgment]." [See 66 Fed. Reg. at 65289; 40 C.F.R. § 125.[81](a)(3)][24]. Once those threshold requirements are met, the facility must use BTA for minimizing AEI. Any other construction improperly reads requirements into the statute that are not in the text and substantially complicates administration.

### AEI OF CWISS

The withdrawal of large quantities of water by CWISs causes AEI in a variety of familiar ways. First, such withdrawal impinges and kills adult fish, eliminating their availability for a number of important functions. Human fishers can no longer catch these fish commercially or recreationally or for sustenance; all opportunities for observation, appreciation, photography, nature study, or research are lost as well as the potential for pecuniary profit associated with any of these uses. Ecologically, the lost fish become unavailable as prey for wildlife higher on the food chain, such as birds, mammals, and larger fish, or to serve as predators for pest insects such as mosquitoes.

[23] 42 U.S.C. § 4332.

[24] For existing facilities, pending the Phase II rule, EPA policy allows a permittee relief from technology requirements in exceptional cases where the costs are "wholly disproportionate to the environmental benefit to be gained." In the Matter of Public Service Company of New Hampshire, et al. (Seabrook Station, Units 1 and 2), 1 EAD 332, 1977 EPA App. Lexis 16, *24-25, June 10, 1977.

Entrainment kills young fish, eggs, larvae, and smaller aquatic life such as plankton by the tens or hundreds of millions. The young fish are thus prevented from maturing to adulthood (where they would provide the benefits discussed immediately above) or even maturing to the next life stage. The vast majority that would have perished before maturity would have contributed to the aquatic ecosystem by consuming prey and ultimately providing fodder for a predator. Because of their death by entrainment, however, they are prevented from doing so. Their contribution to the ecosystem is converted from a gradual, multifaceted process to one in which all of the entrained biota immediately become detritus for decomposers. One result is that energy is transferred down the food chain from higher predators to lower decomposers.

Entrainment and impingement thus disrupts the natural function of the ecosystem, which includes fish, smaller aquatic organisms, insects, birds, mammals, and plant communities. This may broadly alter the nature, structure, and function of the ecosystem, or may damage the sustainability of one or more of its species[25]. Stresses for a particular species may include population decline, reduced prey, more difficulty locating adequate sustenance, or suffering a greater number of pests.

Accurately defining and assessing all of these ever-changing interactions in an ecological community or ecosystem and determining the impact of extraneous manmade and environmental factors changing over time is not feasible[26], especially within the constraints of a permitting proceeding. Apparent stability or robustness in a population may not reflect the impact of significant

[25] In the Phase I Rule, EPA determined that there are:

> multiple types of undesirable and unacceptable adverse environmental impacts, including entrainment and impingement; reductions of threatened, endangered, or other protected species; damage to critical aquatic organisms, including important elements of the food chain; diminishment of a population's compensatory reserve; losses to populations, including reductions of indigenous species populations, commercial fishery stocks, and recreational fisheries; and stresses to overall communities or ecosystems as evidenced by reductions in diversity or other changes in system structure or function.

66 Fed. Reg. at 65292.

[26] EPA concurred with a National Marine Fisheries Service panel conclusion on the difficulty of assessing impact on complex fishery ecosystems:

> As a recent NMFS advisory panel expressed it, "Uncertainty and indeterminancy are fundamental characteristics of the dynamics of complex adaptive systems. Predicting the behaviors of these systems cannot be done with absolute certainty, regardless of the amount of scientific effort invested." Consistent with its own Guidelines for Ecological Risk Assessment, EPA agrees with the conclusions of the NMFS panel that "Given the high variability associated with ecosystems, managers should be cognizant of the high likelihood for unanticipated outcomes."

66 Fed. Reg. at 65293, citing National Marine Fisheries Service Ecosystem Principles Advisory Panel, *Ecosystem-based fishery management: A report to Congress,* 1998.

environmental stresses. A fish population in decline may be bolstered by migrants from other communities, so that the stressors on the community go unnoticed. Also, populations may stabilize at a lower equilibrium level than would be the case absent cooling-water intake mortality. Further, if some percentage of a fish population or community is killed by CWISs, the loss of fish to other causes, both natural and manmade, will aggregate to cause a greater cumulative impact. The EPA based its refusal to define an AEI threshold for the Phase I Rule, in part, on concern that:

> historical overfishing increased the sensitivity of coastal ecosystems to subsequent disturbance, making them more vulnerable to human impact and potential collapse. Based on the long-term record of anthropogenic impacts to coastal ecosystems, their documented degradation, and their potential sensitivity to additional anthropogenic disturbance, as well as the admitted uncertainty associated with managing coastal fishery populations, EPA firmly believes that protective, risk-averse measures are warranted to prevent further declines or collapses of coastal and other aquatic ecosystems. EPA views impingement and entrainment losses to be one of many potential forms of disturbance that should be minimized to avoid further degradation[27].

Thus, an impact that would have been trivial can become significant because power plants consumed the reserve margin that would have allowed the population to withstand the other impact.

### The Conflation of Minimization of AEI with Fisheries Management

Environmental law distinguishes between exploitation of resources for sustenance, such as killing wild organisms for food (hunting and fishing), and collateral or wasteful killing, for example as a by-product of industrial processes. Harvesting of plants and animals for food is fundamental to survival, so the law tolerates the resulting ecological impact (such as reduction in size of wild populations) to a substantial extent. Recreational taking of organisms is also accepted and encouraged in many instances as a social good. Typically, regulation of such activities as hunting and fishing attempts to maximize the availability of prey by balancing the permitted take with the long-term stability

[27] 66 Fed. Reg. at 65293.

of the population[28]. The science of fisheries management focuses on the sustainability of populations, so as to allow fishers and consumers to benefit from a continuous harvest.

A critical basis for industry's approach to section 316(b), and its interpretation of AEI in particular, is the inclusion of BTA regulation within the framework of fisheries management — essentially characterizing entrainment and impingement as fishing. It asserts, "[b]ecause entrainment and impingement are forms of harvesting which (upon conversion to equivalent adults) are analogous to fishing, the methods used by fisheries scientists to evaluate the impacts of proposed harvesting regimes also can be used to evaluate the potential impacts of CWISs"[29]. Despite industry's claim, we do not know of any federal or state governmental body, legislative, executive or administrative, which considers the unnecessary killing of aquatic biota as a socially beneficial harvest. Allocation of scarce fishery resources to cooling-water intake mortality and away from fishers is almost unthinkable in any area of the U.S. with declining or restricted populations and depressed fishing communities.

Nothing in the CWA reflects intent to manage collateral industrial impacts on aquatic ecosystems as a sustainable harvest. Congress's requirement to use the BTA to minimize AEI sets forth a dramatically different protective standard from, for example, the Magnuson Stevens Act provision enacted just 4 years later to allow "on a continuing basis, the optimum yield from each fishery"[30]. Other than the explicit variance for thermal discharges in section 316(a)[31], the CWA and the NPDES program in particular represent a marked Congressional determination to regulate industrial discharges and cooling-water withdrawals through technology-based standards rather than by ecological assessment.

[28] See e.g. Magnuson Stevens Fishery Conservation and Management Act § 301(a)(1); 16 U.S.C. § 1851(a)(1) ("Conservation and management measures shall prevent overfishing while achieving, on a continuing basis, the optimum yield from each fishery for the United States fishing industry.")

[29] UWAG Phase I Comment at p. 66.

[30] 16 U.S.C. § 1851(a)(1).

[31] Despite industry's longstanding insistence, see e.g UWAG Phase I Comment at 16-20, neither the inclusion of cooling-water intake regulation in section 316 nor the legislative history of the CWA indicates Congressional intent to regulate intakes according to the explicit ecologically based variance for thermal discharges in section 316(a). First, the plain language of section 316(b), "best technology available to minimize adverse environmental impact" bears no similarity to the 316(a) variance where "the effluent limitations [are] more than necessary to assure the [protection] and propagation of a balanced, indigenous population of shellfish, fish and wildlife in and on the body of water into which the discharge is to be made…" and does not contemplate such ecological calculation. Instead, the BTA standard much more closely resembles the BTA standards of sections 301 and 306. Moreover, the factors that led Congress to enact the section 316(a) variance for thermal discharges do not apply to fish mortality caused by entrainment and impingement. In the hearings on the CWA Congress heard "extensive and detailed testimony" about the "unique characteristics of heat as a pollutant," including its ability to "dissipate quickly, result in only local and temporary effects and…benefit the environment under circumstances." W. Anderson, II and E. Gotting, *Taken In Over Intake Structures? Section 316(b) of the Clean Water Act*, 26 *Colum. J. Envtl. L.* **1**, 83–86. None of these unique characteristics of thermal pollution are shared by industrial fish kills, particularly on a large scale.

### INDUSTRY'S USE OF POPULATION-BASED AEI MODELS TO AVOID MINIMIZING FISH KILLS

Section 316(b) determinations have typically involved individualized ecological assessment and determination of BTA for each proposed or renewed CWIS. The multiplicity of these individual determinations and the combination of ecological and mathematical/statistical expertise necessary to determine the complex population dynamics for individual species has granted industry a critical strategic advantage because of superior resources in these proceedings. Industry's insistence on some arbitrary threshold of AEI would further tip the scales in its favor by requiring a detailed ecological assessment simply to subject the CWIS to the fundamental minimization requirement in section 316(b).

Industry's most common analytical tools in these individualized technical determinations are density-dependent models of fisheries populations. Cooling-water users have for decades used arguments based on density-dependence to justify the destruction of large numbers of fish and crustaceans via impingement and entrainment at their CWISs. Compensatory density-dependence, in general, is a common natural adjustment in an animal population's birth, death, or survivability rates to maintain equilibrium despite potential increases or decreases in population levels. A population will continue either increasing or decreasing unless at least one of these rates is density-dependent at some life stage. Mathematical models of density-dependent compensation can predict relatively stable populations despite severe anthropogenic mortality.

In many critical cases these models have been misapplied. Typically, their use is based on a generalized assumption of density dependence; regulators rarely require an explanation or demonstration of the actual existence of any pertinent compensation mechanism. Models reflecting compensation due to environmental conditions and periods of relative population abundance may not be appropriate in less abundant circumstances, even for the same species. Nevertheless, industry consultants often utilize models derived from fisheries[32]

[32] In deciding not to rely on population estimates to determine AEI, EPA cited the inability of sophisticated fishery models to reliably forecast even the fishing impacts for which they were derived, especially for populations in decline:

> Despite the availability of state-of-the-art fish population models and considerable experience managing fisheries, NMFS recently classified 34% of their managed fishery stocks as over-utilized. EPA agrees with fisheries experts and resource managers that there is unavoidable uncertainty associated with managing fish populations…EPA and other fishery scientist [*sic*] support the concept of a precautionary approach, particularly when dealing with complex systems, as described below.
>
> EPA recognizes that the limitations of existing population models, including models used to manage fisheries, may be related to our overall limited understanding of the complexity of aquatic ecosystems and the long-term effects of anthropogenic activities.

66 Fed. Reg. at 65293 (citations omitted).

influenced by biological factors and compensation mechanisms that are absent from the populations and life stages damaged by their cooling-water withdrawals. For example, commonly used Ricker curves, originally developed for salmonid fisheries with intense competition for spawning space, reflect compensation based on the surviving adults' greater opportunity to successfully spawn due to increased availability of suitable sites. This mechanism could not effectively compensate for entrainment mortality because it would operate prior to the development of entrainable life stages[33], and in many cases is not even found in the species subject to entrainment[34].

More fundamentally, typical compensation analysis relies on an ecologically baseless concept of "surplus production." It dismisses the ecological value of the tens of millions of fish that are a critical base of the food chain whether or not they grow to adulthood — even though their predators may be populated at far below their historic values. It narrowly focuses on the larval fish only as potential adults for individual species, rather than as forage for small predators in varied parts of the larger food web. By killing off forage, power plants create the very mechanism — food limitation — that is among the most likely to lead to the density dependence they may assert for any particular species[35]. Nevertheless, "the appealing, yet narrow, perspective of the surplus production concept … has allowed us to justify, perhaps blindly, prosecution of fisheries"[36]. Prof. Boreman notes:

[33] EPA noted the inapposite application of standard fishery models to entrainment:

> EPA considered the premises underlying MSY [maximum sustainable yield as developed under the Magnuson-Stevens Act] and the models used by National Marine Fisheries Service (NMFS) to derive MSY. Because the concept of MSY is based on harvesting adult fish, EPA generally questions whether this approach is directly relevant to egg, larvae, and juvenile losses associated with intakes.

66 Fed. Reg. at 65292.

[34] See e.g. *Draft Environmental Impact Statement for State Pollutant Discharge Elimination System Permit Renewal for Bowline Point 1 & 2, Indian Point 2 & 3 and Roseton 1 & 2 Steam Generating Stations,* December 1999, Appendix VI-4-B, relying on Ricker models to estimate the impact of entrainment on Atlantic tomcod young.

[35] In the preamble to the Phase I Rule, EPA repeatedly cited concern about the potential impacts of cooling-water withdrawals on the food chain, i.e., the potential loss of forage for predators. See e.g. 66 Fed. Reg at 65263, fn. 1 and 2, 65264, 65291, 65292. In the context of impingement, EPA concluded:

> EPA does not believe that loss of such forage species should be viewed as having limited importance simply because they have minimal or no commercial or recreational value. From a more holistic, ecological perspective, forage species can have great importance in their role as prey for higher trophic levels, including many commercially and recreationally important fish species. In today's rule, EPA seeks to minimize impingement losses for all affected species.

66 Fed. Reg. at 65295.

[36] J. Boreman, 2000, "Surplus production, compensation, and impact assessments of power plants," *Envtl. Sci. and Policy* 31, 445–446.

> If a “surplus” is being removed by power plant operations, then something else in the ecosystem is being out-competed. Use of surplus production is essentially an allocation issue among competitors for that resource. Do we use it for supporting fisheries, for allowing the population to hedge against bad times, for providing extra sustenance for natural predators, or for supporting other uses of the resource?[37]

Moreover, industry’s compensation theories have no explanation for why fish would have evolved to create long-term surplus production. High fecundity serves to maintain population levels despite multiple, variable stressors, such as climatic, environmental, and other natural causes of mortality. Production of individuals in excess of what is needed to replenish population in any particular year also cushions against intermittent excessive mortality. Destruction of seemingly excess offspring through entrainment could render the surviving class vulnerable to other stressors that it might otherwise have been able to endure; as a result, what constitutes surplus production in one year may be needed the next to counteract changes in environmental conditions that affect cohort survival[38]. Natural selection would have reduced such high fecundity, which places great demands on fish, if it truly exceeded long-term ecological needs. It did not evolve to allow the fish to be killed in large numbers by a power station. In implementing its Phase I Rule, EPA specifically cited the diminution of a population’s potential compensatory reserve due to such mortality as an AEI within the meaning of section 316(b)[39].

Ironically, the presumption of overwhelming compensation for the species of fish entrained by CWISs is the only critical ecological feature that the power industry and their consultants assume is universal, not subject to the requirement of individualized determination. An applicant’s burden of proof, especially for the counterintuitive notion that the destruction of large numbers of fish is “ecologically irrelevant” should typically require greater a greater showing of density dependence than has been typically required to date.[40]

[37] *Id.* at S447.

[38] *Id.* at S446. EPA similarly found that:

> a population’s potential compensatory ability is affected by all stressors encountered within the population’s natural range, including takes attributed to individual or multiple cooling water intake structures. Thus, even if there is little evidence that cooling water intakes alone reduce a population's compensatory reserve, EPA is concerned that the multitude of stressors experienced by a species can potentially adversely affect its ability to recover.

66 Fed. Reg. at 65294.

[39] 66 Fed. Reg. at 65291, 65292

[40] See e.g. New York State regulations for burden of proof in environmental adjudicatory hearings at 6 N.Y.C.R.R. § 624.9(b)(1).

Some of the models utilized by industry make density dependence seem inevitable because they mathematically derive compensatory mechanisms based on almost any input. For example, in the joint permit renewal of three of the power stations on the Hudson River, the applicants relied on a striped bass model based on Beverton-Holt functions, which assume *a priori* strong compensation during early life stages subject to entrainment[41]. The model effectively derives density-dependent results from environmental variation and other nondensity dependent factors, which may well actually control the population. The independent consultant hired by New York State to review the Draft Environmental Impact Statement prepared by the applicant for the permit renewals concluded:

> We believe the striped bass modeling results and conclusions…are unreliable due to limitations of the data, modeling of data and model assumptions. These limitations may cause the DEIS striped bass model to estimate extremely high and counter-intuitive levels of density dependent mortality.…We believe the qualitative effect of the types of errors we discuss in our full report…support an alternative hypothesis of much lower density-dependence (and higher sensitivity to entrainment and impingement) than the results presented in the DEIS for the striped bass population[42].

The indiscriminate use of density-dependent models of fishery population has substantially allowed power generators to eviscerate statutory requirements to minimize AEI. Even where applicants fail to support or inappropriately apply these models, simply understanding their implication requires an extraordinary level of biostatistical sophistication beyond the range of most permitting agencies and members of the public, which typically do not have the resources necessary for effective critical review. Industry's use of such models will typically undergo less scrutiny than in the historic conflict over the Hudson River power plants, and thereby avoid critical questioning even though they may be no more appropriate. As a result, ubiquitous assertions of density dependence, whether overt or implicit in stock-recruitment equations, have effectively allowed applicants to utilize individualized cooling-water intake review to avoid the statute's minimization mandate.

[41] See *Draft Environmental Impact Statement for State Pollutant Discharge Elimination System Permit Renewal for Bowline Point 1 & 2, Indian Point 2 & 3 and Roseton 1 & 2 Steam Generating Stations* Appendix VI-4.

[42] ESSA Technologies Ltd., *Review of the Draft Environmental Impact Statement for SPDES Permits for Bowline Point 1 & 2, Indian Point 2 & 3, and Roseton 1 & 2 Steam Electric Generating Stations*, October 20, 2000, at v.

### AGENCY INTERPRETATION OF AEI

In 1977, EPA issued section 316(b) guidance for regional offices and state regulators[43], defining AEI as follows:

> Adverse aquatic environmental impacts occur whenever there will be entrainment or impingement damage as a result of the operation of a specific cooling water intake structure. The critical question is the magnitude of any adverse impact[44].

This definition properly acknowledges that harm to aquatic life through impingement and entrainment including "damage" to fish, short of death, is AEI. The guidance also recognized the difficulty in assessing the impacts of impingement and entrainment on all species in an ecosystem:

> Adverse environmental impact may be felt by many species in all trophic levels. A species need not be directly affected but nevertheless harmed due to loss of food organisms or other associated organisms in some way necessary for the well-being and continued survival of the population. It is not practicable to study all species that may be directly or indirectly harmed by intake structure operations[45].

The EPA Phase I Rule decisively rejects proposals to restrictively define AEI in terms of identifiable population level impacts. The rule contains no explicit definition, and in the preamble EPA acknowledges that:

> there are multiple types of adverse environmental impact including impingement and entrainment; reductions of threatened, endangered, or other protected species; damage to ecologically critical aquatic organisms, including important elements of the food chain; diminishment of a population's potential compensatory reserve; losses to populations, including reductions of indigenous species populations, commercial fishery stocks, and recreational fisheries; and stresses to overall communities or ecosystems as evidenced by reductions in diversity or other changes in system structure or function[46].

[43] U.S. Environmental Protection Agency, Office of Water Enforcement, Permits Division, Industrial Permits Branch, *Guidance for Evaluating the Adverse Impact of Cooling Water Intake Structures on the Aquatic Environment: Section 316(b), P.L. 92-500*, Washington, D.C., May 1, 1977.

[44] *Id*. at pp. 11, 15 (the quoted text appears on both cited pages).

[45] *Id*. at p. 15.

[46] 66 Fed. Reg. at 65291.

The EPA further clarifies:

> The Agency has long maintained that adverse environmental impact from cooling water intake structures must be minimized to the fullest extent practicable, even in cases where it can be demonstrated that the requirement applicable under section 316(a) is being met [*i.e.* that the projection and propagation of a balanced, indigenous population of shellfish, fish, and wildlife is assured in and on the receiving waterbody]. Thus, the objective of section 316(b) includes population effects but is not limited to those effects[47].

Thus, despite pointed advocacy from industry commenters[48], the rule for new facilities contains no threshold, other than the 2 million gal/day *de minimus* withdrawal, before the section 316(b) minimization requirement applies.

In most states, the federal CWA NPDES program, including section 316(b), is implemented by state regulatory agencies pursuant to a delegation agreement with the EPA. State agencies, therefore, typically determine the BTA for minimizing AEI and issue NPDES permits that incorporate those requirements. For new facilities, they will administer the Phase I Rule; for existing plants they will continue to determine requirements on a site-specific basis until EPA promulgates the Phase II Rule. It is highly significant, then, that a majority of states commenting on the EPA's proposed definitions of AEI recognize that any impingement and entrainment constitutes AEI. During the public comment period on the Phase I Rule, New York, New Jersey, Pennsylvania, Michigan, and Alaska, as well as the Atlantic State Marine Fisheries Commission, each recommended that if EPA were to define AEI, it should adopt the proposed third definition because in their experience it is accurate and workable[49].

The New York State Department of Environmental Conservation (NYSDEC), which has had extensive experience with large-scale impingement and entrainment on the Hudson River, properly recognizes that the killing of any fish or other aquatic biota negatively impacts the ecosystem, and that no more fish than necessary should be allocated to death by impingement and entrainment:

> Our program considers the death of any fish at or through a cooling water intake to be an "adverse impact". In the past the Department has stated that it is not appropriate to allocate a component of the

[47] 66 Fed. Reg. at 65292.

[48] See e.g. 66 Fed. Reg. at 65291-65295.

[49] EPA's proposed third definition was "AEI would be deemed to occur whenever aquatic organisms are impinged or entrained as a result of the operation of a cooling water intake." 65 Fed. Reg. at 49074-49075.

> public fish and wildlife resource to electric energy generators or other cooling water intakes. This pragmatic approach to defining adverse impact avoids the alternative of trying to determine the incremental impact of each intake upon the populations of dozens of affected species. That is, it avoids potentially endless, expensive studies that usually yield ambiguous or debatable results. The results are often debatable because it is impossible to identify, measure, and attribute the impact of each the many variables affecting populations on each of the impacted species[50].

Likewise, the State of New Jersey Department of Environmental Projection (NJDEP) asserted that:

> this third alternative [definition of AEI as impingement and entrainment] is the only practical alternative and should be adopted… This is the same definition that the Department currently uses in applying Section 316(b) policies for existing facilities. *The Department considers the death of any fish at or through a cooling water intake structure to be an "adverse impact" which must be minimized under Section 316(b). This position makes sense and simplifies an already complex analysis.* State agencies and permitting authorities could engage in a debate for years as to the population measure of a given fish species, let alone many fish species. The results of biological population studies and modeling can be very subjective because it is difficult to identify, measure, and attribute the impact of each of the many variables…affecting populations of the impacted species. Rather than engage in this kind of biological debate, time and resources would be better spent focusing on the magnitude of the impingement and entrainment losses in relation to the costs and benefits of implementing various technologies to avoid or minimize the impact. This focus is appropriate for section 316(b) which the Department feels is a technology-driven provision[51].

And the Michigan Department of Natural Resources (MDNR)

> support[s] the third alternative, which is a similar approach taken by the State of New York that defines adverse environmental impact as

[50] New York State Department of Environmental Conservation, Division of Fish, Wildlife, and Marine Resources, Clean Water Act section 316(b), S*tatement provided to U.S. Environmental Protection Agency, June 29, 1998: Public Meeting to Discuss Adverse Environmental Impacts resulting from Cooling Water Intake Structures*, at p. 1. In addition, NYSDEC states that the goal of CWIS regulation should be zero mortality. This is consistent with minimizing AEI.

[51] *State of New Jersey, Department of Environmental Protection. Letter from Dennis Hart, Assistant Commissioner, to USEPA dated November 9, 2000* at p. 4 [emphasis added].

> any impingement or entrainment of aquatic organisms....The State of Michigan has experienced considerable inaction in the adoption of technology because of disagreement among power producers and agency biologists if operation of the facility is causing adverse impact. The adoption of the new language would make the definition of adverse impact very clear and ultimately better protect the aquatic resource[52].

### CONCLUSION

Industry's insistence on a separate definition of AEI is a stratagem to create a significant procedural and technical roadblock to effective regulation of CWISs. Such a definition would set an inappropriate threshold before regulation could even take place, and would ironically complicate the task of determining BTA to minimize AEI. It would also confer substantial advantages on industrial applicants due to their superior resources. AEI includes any aquatic mortality due to impingement and entrainment, and EPA should by regulation require the BTA to minimize such impact. EPA properly refused to burden its Phase I new facility rule with this unnecessary threshold determination and should similarly eschew it in promulgating the Phase II Rule for existing facilities.

CWA § 402.

Draft Environmental Impact Statement for State Pollutant Discharge Elimination System Permit Renewal for Bowline Point 1 & 2, Indian Point 2 & 3 and Roseton 1 & 2 Steam Generating Stations.

ESSA Technologies Ltd. (2000). Review of the Draft Environmental Impact Statement for SPDES Permits for Bowline Point 1 & 2, Indian Point 2 & 3, and Roseton 1 & 2 Steam Electric Generating Stations. October 20.

Grad, F. (1973). *Treatise on Environmental Law.* M. Bender, New York.

Hines, N. W. (1968). Controlling industrial water pollution: Color the problem green. *9 B.C. Indus. and Comm. L. Rev.* **553**.

Hill, T. (2000). Letter from NEFMC Chairman Thomas Hill to U.S. Environmental Protection Agency, November 17.

Magnuson Stevens Fishery Conservation and Management Act § 301(a)(1).

Michigan Department of Natural Resources. (2000). Letter to USEPA dated November 7, 2000.

Nagle, D. G. and Morgan, J. T. (2000). *Environ. Sci. Policy* **3**.

New York State Department of Environmental Conservation, Division of Fish, Wildlife, and Marine Resources, Clean Water Act section 316(b). (1998). Statement provided to U.S. Environmental Protection Agency, June 29: Public Meeting to Discuss Adverse Environmental Impacts resulting from Cooling Water Intake Structures.

*New York Times*, p. 41, August 16, 1972 (Abstr.).

*New York Times*, p. 77, March 1, 1972 (Abstr.).

*New York Times*, p. 94, May 24, 1972 (Abstr.).

NOAA. (2000). Comments on the Proposed Rule for Cooling Water Intake Structures for New Facilities, provided to U.S. EPA on December 18.

*Random House Webster's College Dictionary*. (1999).

Senate Committee on Public Works. (1973). A Legislative History of the Water Pollution Control Act Amendments of 1972, 93d Congress, 1st Session, at 196-197.

State of New Jersey, Department of Environmental Protection. (2000). Letter from Dennis Hart, Assistant Commissioner, to U.S. Environmental Protection Agency, November 9.

U.S. Environmental Protection Agency, Decision of the General Counsel, EPA GCO 41. (1976). *In the Matter of: Carolina Power & Light Company (Brunswick Steam Electric Plant)*. June 1.

U.S. Environmental Protection Agency. (1977). *In the Matter of Public Service Company of New Hampshire, et al. (Seabrook Station, Units 1 and 2)*. 1 EAD 332, App. Lexis 16, *24-25.

U.S. Environmental Protection Agency. (1977) Development Document for Best Technology Available for the Location, Design, Construction and Capacity of Cooling Water Intake Structures for Minimizing Adverse Environmental Impact. *Virginia Electric and Power Company v. Costle* (VEPCO). 566 F.2d 446 (4th Cir.).

U.S. Environmental Protection Agency, Office of Water Enforcement, Permits Division, Industrial Permits Branch. (1977). Guidance for Evaluating the Adverse Impact of Cooling Water Intake Structures on the Aquatic Environment: Section 316(b), P.L. 92-500. Washington, D.C.

16 U.S.C. § 1851(a)(1).

33 U.S.C. § 1326(b).

33 U.S.C. §§ 1312, 1313.

40 C.F.R § 125.81(a)(3).

40 C.F.R Parts 402–699.

40 C.F.R. Parts 130–131.

42 U.S.C. § 4332.

6 N.Y.C.R.R. § 624.9(b)(1).

65 Fed. Reg. 49059 (August 10, 2000).

66 Fed. Reg. 28853 (May 25, 2001).

66 Fed. Reg. 65256 (December 18, 2001).

### BIOSKETCHES

**Reed W. Super** is a Senior Attorney at Riverkeeper, Inc., a not-for-profit environmental organization based in Garrison, New York. Riverkeeper is dedicated to preserving the ecological integrity of the Hudson River, and Riverkeeper's National Fisheries and Power Plant Project focuses on the aquatic impacts of cooling water withdrawals. Mr. Super obtained his J.D. and M.B.A. degrees from the University of Virginia in January 1992, and has a BA (1985) from Duke University. Mr. Super practices, teaches, and writes about environmental law.

**David K. Gordon** is also a Senior Attorney at Riverkeeper, and has served there since 1990. Mr. Gordon has a J.D. from the University of Wisconsin Law School (1986), an LL.M. in Environmental Law from Pace University Law School, and a B.S. in economics from Binghamton University. He currently concentrates on reducing impacts to the Hudson River from power plants and other industrial facilities. Prior to this, he served as Reservoirkeeper and helped negotiate the landmark $1.4 billion 1997 agreement to protect the New York City watershed with federal, state, city, and upstate municipal officials. He is also a member of the Town of Lloyd Planning Board and the Vice President of the Hudson Valley Rail Trail Association.
